# Occurrence and characterization of plasmids carrying *tmexCD1*-*toprJ1*, *bla*_DHA-1_, and *bla*_CTX-M-127_, in clinical *Klebsiella pneumoniae* strains

**DOI:** 10.3389/fcimb.2023.1260066

**Published:** 2023-10-13

**Authors:** Ying Qu, Wenji Wang, Qinhong Lu, Jihai Qiu, Dongguo Wang, Liman Ma

**Affiliations:** ^1^ Department of Clinical Medicine Laboratory, Taizhou Municipal Hospital Affiliated with Taizhou University, Taizhou, Zhejiang, China; ^2^ School of Life Sciences, Taizhou University, Taizhou, Zhejiang, China; ^3^ Department of Clinical Medicine Laboratory, Ningbo Medical Center Li Huili Hospital, Ningbo, Zhejiang, China; ^4^ Department of Infectious Diseases, Taizhou Municipal Hospital Affiliated with Taizhou University, Taizhou, Zhejiang, China; ^5^ Department of Central Laboratory, Taizhou Municipal Hospital Affiliated with Taizhou University, Taizhou, Zhejiang, China; ^6^ School of Medicine, Taizhou University, Taizhou, Zhejiang, China

**Keywords:** *K. pneumoniae*, F4_plasmid pA, F4_plasmid pB, tigecycline resistance, multidrug resistance, tmexCD1-toprJ1

## Abstract

**Objective:**

Today, the emergence of *Klebsiella pneumoniae* with the *tmexCD1-toprJ1* gene cassette in patients has presented a significant clinical challenge.

**Methods:**

To present the detailed genetic features of the *tmexCD1-toprJ1* gene cassette of *K. pneumoniae* strain F4_plasmid pA, the whole bacterial genome was sequenced by Illumina and nanopore platforms, and mobile genetic elements related to antibiotic resistance genes were analyzed with a series of bioinformatics methods.

**Results:**

*K. pneumoniae* strain F4 was determined to be a class A+C beta-lactamase, and was resistant to routinely used antibiotics, especially tigecycline, because of the *oqxAB* gene localized on the F4_chromosome and *tmexCD1-toprJ1* on F4_plasmid A. After plasmid transfer assays, the F4_plasmid pA or F4_plasmid pB could be recovered with an average conjugation frequencies of 3.42×10^-4^ or 4.19×10^-4^. F4_plasmid pA carried *tmexCD1-toprJ1* and *bla*_DHA-1_ accompanied by genetic intermixing of Tn*As1*, Tn*5393*, Tn*As3*, and In641, while F4_plasmid pB, bearing *bla*_CTX-M-174_, had structural overlap of Tn*As3* and In641.

**Conclusions:**

We suggested that plasmids carrying *tmexCD1- toprJ1* might be strongly related to IS*26*-integrated loop intermediates. This study showed that due to the structural evolution of F4 and related strains, their resistances were so strong that effective antibiotics were virtually unavailable, therefore their spread and prevalence should be strictly controlled.

## Introduction

In recent years, a novel plasmid-mediated resistance-nodule-division (RND) efflux pump, *tmexCD1*-*toprJ1*, has emerged and been widely characterized from patient, animal, and food samples of *Klebsiella pneumoniae* (Lv et al., 2020). This novel multidrug resistance plasmid gene cluster, *tmexCD1-toprJ1*, was first reported in 2020 from animal-derived *K. pneumoniae* in China, exhibiting resistance or reduced susceptibility to several classes of antibiotics, including cephalosporins, phenolics, quinolones, and tetracyclines, and conferring resistance to the last-line antibiotics tigecycline and eravacycline (Lv et al., 2020; Wang CZ et al., 2021; Ghaheh et al., 2022).

The chromosomal or plasmid-encoded RND family *tmexCD1*-*toprJ1* expresses multidrug resistance (MDR) in Gram-negative bacteria (Li et al., 2015; Du et al., 2018), usually requiring the action of three gene products to be effective. Homologous transfer of the entire gene cluster encoding the RND-type tripartite drug efflux pump from chromosome to plasmid has been rarely reported thus far (Tauch et al., 2003; Li et al., 2015). The tripartite efflux system formed by the RND pump can directly export antibiotics outside the cell (Li et al., 2015), but only when regulatory genes (such as *tnfxB*, *araC*, or *tetR*) are efficiently expressed intracellularly does this lead to MDR in most clinically pathogenic bacteria carrying *tmexCD1*-*toprJ1* (Salehi et al., 2021).

Tigecycline is one of the limited options for the treatment of infections caused by MDR gram-negative bacteria, particularly carbapenem-resistant Enterobacteriaceae (De Oliveira et al., 2020). However, two novel plasmid-mediated mechanisms for tigecycline resistance have recently been determined, including tigecycline-resistance variants of the *tet(X), tet(A), tet(K)*, and *tet(M)* genes (Linkevicius et al., 2016; Liu et al., 2016; He et al., 2019; Lv et al., 2020; Xu et al., 2021), and the RND efflux pump gene cluster, *tmexCD1*-*toprJ1* (Lv et al., 2020). The *tet* variants are rarely discovered in *K. pneumoniae* (He et al., 2019; Wang et al., 2019), but *tmexCD1*-*toprJ1* and its variants are increasingly found in *K. pneumoniae* (Lv et al., 2020; Sun et al., 2020), and severe variants can lead to high mortality in patients (Yang et al., 2021; Wang et al., 2023). The emergence of tigecycline resistance from plasmids with *tmexCD1*- *toprJ1* is highly disseminated and poses a significant clinical challenge. IS26-mediated mobility of the *tmexCD1-toprJ1* plasmid may result in rapid and widespread dissemination of *tmexCD1-toprJ1* among Gram-negative bacteria (Wan et al., 2021).

Transposons could carry multiple drug-resistant genes in different plasmids, especially, Tn*3* played a crucial role in the evolution of drug-resistant plasmids in Enterobacteriaceae (Algarni et al., 2023). And Integrons could be integrated into antibiotic resistance gene cassettes with multiple IS elements or with transposons to form complex structures containing multiple resistance genes (Algarni et al., 2023), that contributed to the widespread spread of drug-resistant genes among bacteria (Algarni et al., 2022).

In this study, we compared and analyzed the MDR region of the F4 plasmids bearing *tmexCD1*-*toprJ1*, *bla*_DHA-1_, and *bla*_CTX-M-127_ accompanied by intermingling of In641 or In553 with Tn*As1*, Tn*5393*, and/or Tn*As3* with those of related plasmids, characterized the structure of the plasmids.

## Materials and methods

### Bacterial strains and sequencing of the 16S rRNA gene

*K. pneumoniae* strain F4 was isolated from a sputum sample of a patient in Taizhou Municipal Hospital affiliated with Taizhou University in 2019. EC600 and *Escherichia coli* DH5α (TaKaRa, Dalian, China) were employed as hosts for cloning. In order to verify the strain F4 belonged as *K. pneumoniae*, the nearly complete 16S rRNA gene of the strain was amplified by PCR using the following primers: 5’-AGAGTTTGATYMTGGCTCAG-3’ (forward) and 5’-TACCTTGTTACGACTT-3’ (Y, T or C; M, A or C) (reverse). The length of the amplicon was about 1500 bp (Frank et al., 2008). The Taq enzyme was a 3:1 mixture of Fermentas Taq : Pfu ThermoFisher Scientific, Burlington, VT, USA), and the 30 ml reaction consisted of 1.5 U of enzyme. Amplification was performed using a temperature program, including initial denaturation at 94°C for 3 min, 30 cycles of denaturation at 94°C for 40 s, annealing at 50°C for 40 s, extension at 72°C for 1 min, and final extension at 72°C for 5 min. The PCR products were identified by bidirectional sequencing.

### Experiments of conjugal transfer and plasmid transfer

#### Conjugation experiments

Based on a previously reported (Chen et al., 2015), conjugation experiments were performed in lysogeny broth (LB) with the strain EC600 as the recipient and with the strain F4 as the donor strains. Donor and recipient cells in logarithmic phase (0.5 mL of each) were added to 4 mL of fresh LB, which was followed by incubation at 35°C for 18–24 h without shaking. The transconjugants were selected on trypticase soy agar (TSA) plates containing 10 μg/L of rifampicin and 0.02 μg/L of imipenem.

#### Plasmid-electroporation assays

Transformation experiments were undertaken using *E. coli* DH5α electroporated cells as recipient cells for plasmid electroporation. Conjugation frequency was calculated as the number of transconjugates per initial donor cell. To prepare electrocompetent cells, bacteria were grown to OD600 = 0.5-0.6 and precipitated by centrifugation at 4°C. Two rounds of washes and centrifugation (6,000 rpm) were performed at 4°C with 1 vol milliQ water, ending with 1/50 volume 10% glycerol. Cells were resuspended in 1/500 vol 10% glycerol and aliquoted into 50 mL samples. Aliquots were frozen on dry ice, stored at -70°C and set aside. Aliquots were mixed with less than 10 ng of DNA in a 0.2 cm cuvette (Bio-Rad, California, USA) and then electrically pulsed (2.5 kV, 25 mF and 200 Ω) in a MicroPulser (Bio-Rad, California, USA). Electroporated cells were added to 1 mL of LB and incubated at 37°C with shaking for antibiotic expression. After incubation, the cells were cultured on antibiotic-containing medium. When plasmids from the strain F4 were used in electroporators, it was also selected appropriately with 10 μg/L of rifampicin and 0.02 μg/L of imipenem.

### Antimicrobial susceptibility test

Bacterial resistance was detected by BioMerieux VITEK2 (MICs value) and disk diffusion test (mm value) (**Table 1**), the results were determined in accordance with the 2022 Clinical and Laboratory Standards Institute (CLSI) Guidelines (CLSI, 2022). Twenty-two antibiotics and antibiotics + enzyme inhibitors (**Table 1**) were detected, and *E. coli* ATCC 25922 was used as the quality control strain.

**Table 1:** MICs and genetic profiles of *K. pneumoniae* F4 (ST15).

Antimicrobial agents	MIC (mg/L)	Mechanism of resistance/ location of resistance gene
Aminoglycoside		
Amikacin	≥16	aac(6’)-Ib/aadA2/aadA1/aac(3’)-IV/aph(4’)-Ia/aph(6’)-Id/ aph(4’)-Ia/aph(3’)-Ia/rmtB/aph(3’)-Ib/tmexCD1-toprJ1 (Plasmid A), aadA16/aac(6’)-Ib-cr (Plasmid B)
β-lactams		
Urtapenem	≥8	bla_DHA-1_ (Plasmid A)/bla_CTX-M-174_ (Plasmid B)
Imipenem	≥2	bla_DHA-1_ (Plasmid A)/bla_CTX-M-174_ (Plasmid B)
Aztreonam	≥32	bla_DHA-1_ (Plasmid A)/bla_CTX-M-174_ (Plasmid B)
Cefepime	≥32	bla_DHA-1_ (Plasmid A)/bla_CTX-M-174_ (Plasmid B)
Cefotaxime	≥64	bla_DHA-1_ (Plasmid A)/bla_CTX-M-174_ (Plasmid B)
Ceftazidime	≥64	bla_DHA-1_ (Plasmid A)/bla_CTX-M-174_ (Plasmid B)
Ceftriaxone	≥64	bla_DHA-1_ (Plasmid A)/bla_CTX-M-174_ (Plasmid B)
Cefuroxime	≥64	bla_DHA-1_ (Plasmid A)/bla_CTX-M-174_ (Plasmid B)
Amoxicillin/ Clavulanic acid	≥32	bla_DHA-1_ (Plasmid A)/bla_CTX-M-174_ (Plasmid B)
Cefoperazone/ Sulbactam	≥64	bla_DHA-1_ (Plasmid A)/bla_CTX-M-174_ (Plasmid B)
Cefoperazone/ Avibactam (30/20 µg)	6^*^	bla_DHA-1_ (Plasmid A)/bla_CTX-M-174_ (Plasmid B)
Cephamycin		
Cefoxitin	≥64	bla_DHA-1_ (Plasmid A)/bla_CTX-M-174_ (Plasmid B)
Fluoroqinolones		
Levofloxacin	≥8	tmexCD1-toprJ1/qnrB4 (Plasmid A), qnrB2 (Plasmid B), qnrB2 (Plasmid C), oqxB/oqxA (Chromosome)
Ciprofloxacin	≥4	tmexCD1-toprJ1/qnrB4 (Plasmid A), qnrB2 (Plasmid B), qnrB2 (Plasmid C), oqxB/oqxA (Chromosome)
Tetracycline		
Tetracycline	≥64	tmexCD1-toprJ1 (Plasmid A)/tet(D) (Plasmid B)
Glycylcycline		
Tigecycline	≥8	oqxAB (Chromosome), tmexCD1-toprJ1 (Plasmid A)
Phenicol		
Chloramphenicol	≥64	cmlA1 (Plasmid A), floR (plasmid B)
Sulfonamide		
Trimethoprim- sulfamethoxazole	≥320	sul1/sul3 (Plasmid A), dfrA27/sul1 (plasmid B), sul1 (plasmid C)
Disinfecting agent and antiseptic		
–	–	qacH2 (Plasmid A)

^*^Disc diffusion method.

## Detection of classes A, B, C and D beta-lactamases

### Dual inhibitor diffusion synergy test

Amoxicillin/clavulanic acid (AMC, 20/10 µg) was placed in the center of the plate, and cefotaxime (CTX, 30 µg), cefepime (FEP, 30 µg), ceftriaxone (CRO, 30 µg), and cloxacillin (CXC, 200 µg) were placed around the AMC, with CTX and AMC spaced 25 mm apart and the rest spaced 20 mm apart. CXC was obtained from MW & E, UK, and the others were from Oxiod, UK.

Interpretation of test results: AMC was synergistic with CRO or CTX, indicating the sample was positive for extended-spectrum beta lactamases (ESBLs); AMC was not co-operative with CRO or CTX but was in synergy with FEP, and thus positive for ESBLs and AmpC; and CXC co-interacted with CRO or FEP, showing a positive result for AmpC enzyme.

#### AmpC enzyme confirmatory test

Using a modified enzyme extraction three-dimensional test, colonies to be tested were picked and incubated overnight on blood plates according to the literature (Coudron et al., 2000). To make a bacterial suspension with 0.5 McFarland turbidity, 25 µL of bacterial suspension was inoculated into 6 mL trypsin-digested soy broth, incubated overnight on a constant temperature shaker at 35°C at 200 r/min, and centrifuged at 4°C at 4000 r/min for 20 min. The supernatant was discarded, the precipitate was repeatedly freeze-thawed five times at −80°C, and 1.5 mL of 0.01 mol/L phosphate buffer (pH 7.0) was added, vortexed, and mixed. The supernatant was then centrifuged at 14,000 r/min for 2 h at 4°C, thus obtaining the enzyme extract.

Based on the standard paper diffusion method, 0.5 mL of *E. coli* ATCC25922 was applied to a Mueller-Hinton (MH) agar plate. Cefoxitin (FOX, 30 μg) was placed in the center of the plate, a slit was cut radiologically from inside to outside at 5 mm from the edge of the paper plate with a sterile scalpel, and 40 μL of crude enzyme extract was added to the slit from inside to outside with a microsampler to avoid the enzyme solution overflowing the slit, then incubated overnight at 35°C. If an expanded long bacterial area appeared at the junction of the slit and the inhibition circle, it was judged as a positive three-dimensional test, meaning the sample was AmpC-enzyme positive.

#### Confirmation test for ESBLs

The differences in the diameter of the inhibition circles between ceftazidime (CAZ, 30 µg), ceftazidime/clavulanic acid (30/30 µg), and cefotaxime/clavulanic acid (30/30 µg) were determined as a positive result for ESBLs when the difference in the diameters of the inhibition circles of either group of drugs was ≥5 mm, based on the 2022 CLSI Guidelines (CLSI, 2022).

#### Classes B and D beta-lactamases tests

The modified carbapenem inactivation method (mCIM) and modified carbapenem inactivation + EDTA (eCIM) methods recommended by CLSI (CLSI, 2022) were performed for the detection of metallo-β-lactamases. And the increase of the zone of inhibition by ≥5 mm of eCIM *vs*. mCIM was interpreted as a positive result for metallo-β-lactamases (class B carbapenemase).

There was no definitive report of a class D carbapenemase phenotypic assay, which could be confirmed by a process of elimination; if the strain was not inhibited by a class A or B inhibitor, it definitely belonged to class D carbapenemase (Class D beta-lactamase).

#### Sequencing and sequence assembly

Genomic sequencing of strain F4 was performed on the third-generation PacBio Sequel II platform (Pacific Biosciences, CA, USA) and the second-generation sequencing Illumina NovaSeq 6000 platform (Illumina, San Diego, USA) using a DNA library with an average size of ~15 kb (range 10 kb to 20 kb), and on a small fragment libraries ~400 bp (range 150 bp to 600 bp). To improve the reliability of data processing, raw data from PacBio Sequel II were trimmed to obtain the high-quality clean reads (clean data) by Canu v2.2 (https://github.com/marbl/canu). The paired-end short Illumina reads were “*de novo*” assembled using Unicycler v0.4.5 (https://github.com/rrwick/Unicycler) or SPAdes v3.15.3 (https://github.com/ablab/spades). The sequence corrections at the post-assembly level were performed using Pilon v1.24 software (https://github.com/broadinstitute/pilon) based on second-generation sequencing reads. Finally, accurate DNA sequences in the study were obtained.

#### Sequence annotation and comparison in detail

Open reading frames (orfs) and pseudogenes were predicted using *RAST2.0* (Brettin et al., 2015), *BLASTP/BLASTN* (Boratyn et al., 2013), *UniProtKB/Swiss-Prot* (Brettin et al., 2015), and *RefSeq* databases (Brettin et al., 2015). Resistance genes, mobile elements, and other features were annotated using online databases such as *CARD 2023* (Alcock et al., 2023), *ResFinder4.0* (Bortolaia et al., 2020), IS*finder* (Siguier et al., 2006), *INTEGRALL* (Moura et al., 2009), and the *Tn* Number Registry (Tansirichaiya et al., 2019). Clonal MLST was determinated using *MLST 2.0* (https://cge.food.dtu.dk/services/MLST/) and *BacWGSTdb 2.0* (Feng et al., 2021). *MUSCLE 3.8.31* (Edgar, 2004) and BLASTN were used for multiple and pairwise sequence comparisons such as F4_plasmid pA, F4_plasmid pB, and F4_plasmid pC with their closely related plasmids, respectively. Circos plot of plasmids were drawn with CGView (Stothard et al., 2019). All plasmids comparison figures were created by the R pacakge genoPlotR v0.8.11 sofware (http://genoplotr.r-forge.r-project.org/) and edited using Inkscape v0.48.1 (https://inkscape.org/en).

#### Nucleotide sequence accession numbers

The sequences of the F4_chromosome, F4_plasmid pA, F4_plasmid pB, and F4_plasmid pC were deposited on the GenBank database with the accession numbers of CP090397.1, OM144974.1, OM144975.1, and OM144976.1, respectively (**Table 2**). The GenBank accession numbers of the related plasmids compared with F4_plasmid pA and F4_plasmid pB were also shown in **Table 2**.

**Table 2:** Profiles of the *K. pneumoniae* plasmids studied in the paper.

No.	Plasmid	Isolate source	Type	Size (kb)	GC%	status	Accession no.
1	F4_plasmid pA	Patient’s urine (Taizhou, China)	IncFIB- IncHI1B	276.487	46.92	Complete	OM144974.1
2	pMH15-269M_1	Patient’s blood (Viet Nam)	IncFIB	288.040	46.67	Complete	AP023338.1
3	pHN111RT-1	Sewage (Guangzhou, China)	IncFIB	281.670	47.06	Complete	MT647838.1
4	pSZP4-9-2-tmexCD	Pork (Shenzhen, China)	IncFIB	274.231	46.89	Complete	CP075257.1
5	P1-tmexCD	Poultry farms (Beijing, China)	IncFIB	280.953	46.86	Complete	OL348377.1
6	F4_plasmid pB	Patient’s urine (Taizhou, China)	IncFIA	79.722	51.65	Complete	OM144975.1
7	pCTXM27_020046	Patient (Yibin, China)	IncFIA	74.010	51.68	Complete	CP028782.2
8	Plasmid L99-05	Swine ropharyngeal (Guangzhou, China)	IncFIA	75.320	51.65	Complete	CP063211.1
9	p19110124-2	Swine Anal swab (Zhengzhou, China)	IncFIA	73.978	51.71	Complete	CP064179.1

## Results

### Antimicrobial susceptibility test, enzymatic properties, and transferable features

The strain was confirmed to be *K. pneumonia* by BLAST of the 16S rRNA and genomic sequences and average nucleotide homology analysis. MICs for the drug susceptibility test of strain F4 were shown in **Table 1**; Results for the drug susceptibility test of BioMerieux VITEK2 without MICs using the paper diffusion method, e.g., cefoperazone/avibactam, were also listed in **Table 1**. Enzyme characterization was confirmed to be class A+C beta-lactamases. The MLST of the strain F4 sequence was analyzed as ST15 using *MLST 2.0* and *BacWGSTdb 2.0*.

After bacterial conjugative transfers and electroporation assays, the transconjugants integrating the F4_plasmid pA or F4_plasmid pB could be recovered with an average conjugation frequencies of 3.42×10^-4^ or 4.19×10^-4^, respectively. However, F4_plasmid pC failed in multiple plasmid transfer experiments.

### Overview of drug resistance genes for F4_chromosome, F4_plasmid pA, F4_plasmid pB, and F4_plasmid pC

**Figure 1:**
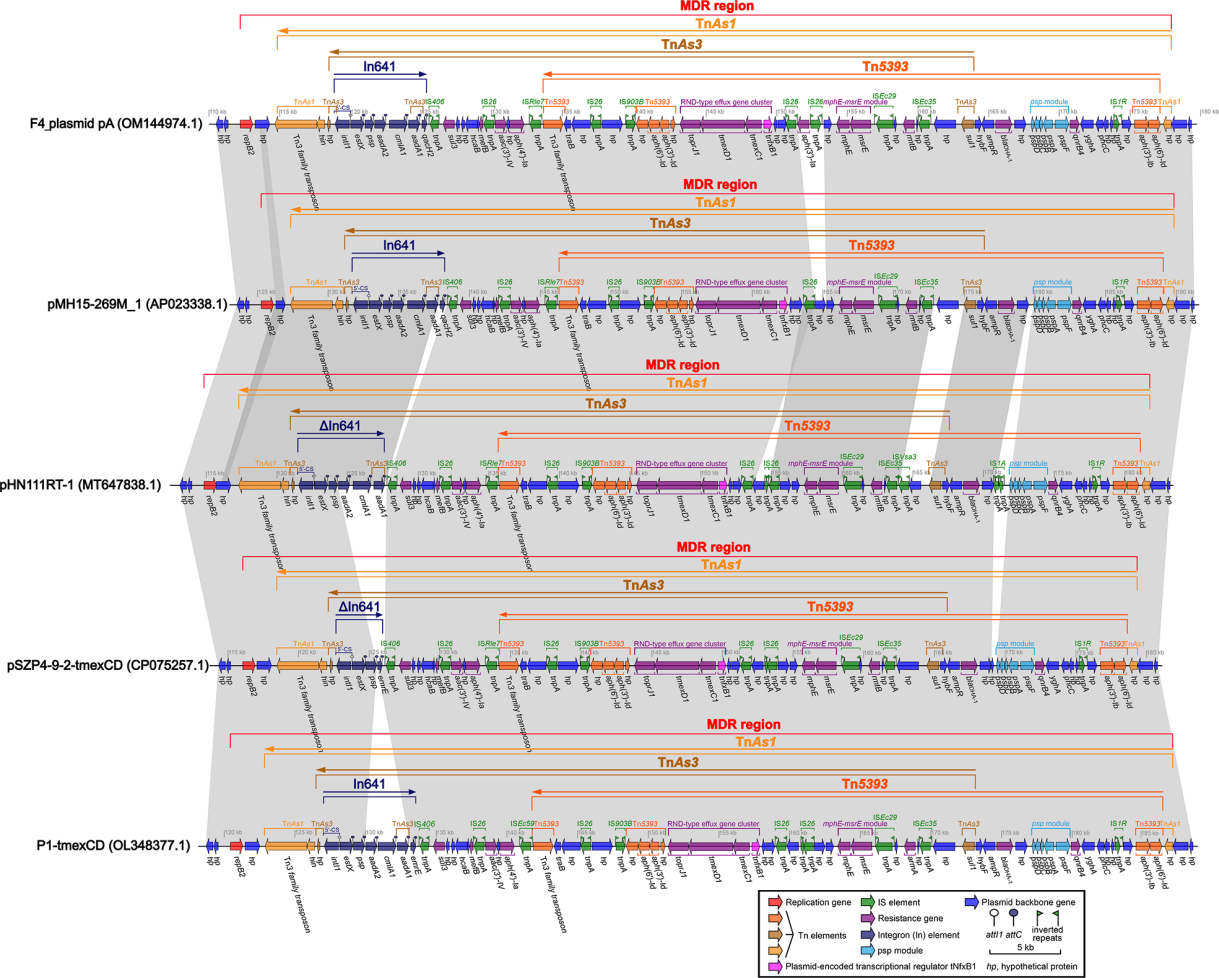
Comparison of F4_plasmid pB with related plasmids pCTXM27_ 020046, Plasmid L99-05, and p19110124-2. The shadow represents > 95% identity, while light blue represents the positive direction, and light pink refers to the opposite direction. The figure was created by the R package genoPlotR v0.8.11 software (http://genoplotr.r-forge.r-project.org/).

One F4_chromosome and three plasmids, including F4_plasmid pA, F4_plasmid pB, and F4_plasmid pC, were identified in strain F4. The F4_chromosome was approximately 5.25 Mb in length and only carried four antibiotic resistance genes, *oqxB*, *oqxA*, *bla*_SHV-106_, and *bla*_CTX-M-27_ (**Table 1**, **Figure S1**). F4_plasmid pA is a complete plasmid with a length of about 276.5 kb, harboring 22 different categories of drug resistance genes (**Table 1**, **Figure S2**). F4_plasmid pB was also a complete plasmid with a length of about 79.7 kb, carrying nine different types of drug resistance genes (**Table 1**, **Figure S2**). The length of F4_plasmid pC was 5.721 kb, but it belonged to a plasmid fragment with only quinolone (*qnrB2*) and sulfonamide (*sul1*) resistance genes (**Table 1**, **Figure S2**). F4_plasmid pC shared identity with the back-end sequence of F4_plasmid pB and a portion of the sequence before In553 of plasmid L99-05, but in the opposite directions (**Figure 1**).

### Comparison of F4_plasmid pA with closely related plasmids pMH15-269M_1, pHN111RT-1, pSZP4-9-2-tmexCD, and P1-tmexCD, carrying *tmexCD1*-*toprJ1*, *bla*_DHA-1_, and In641

**Figure 2:**
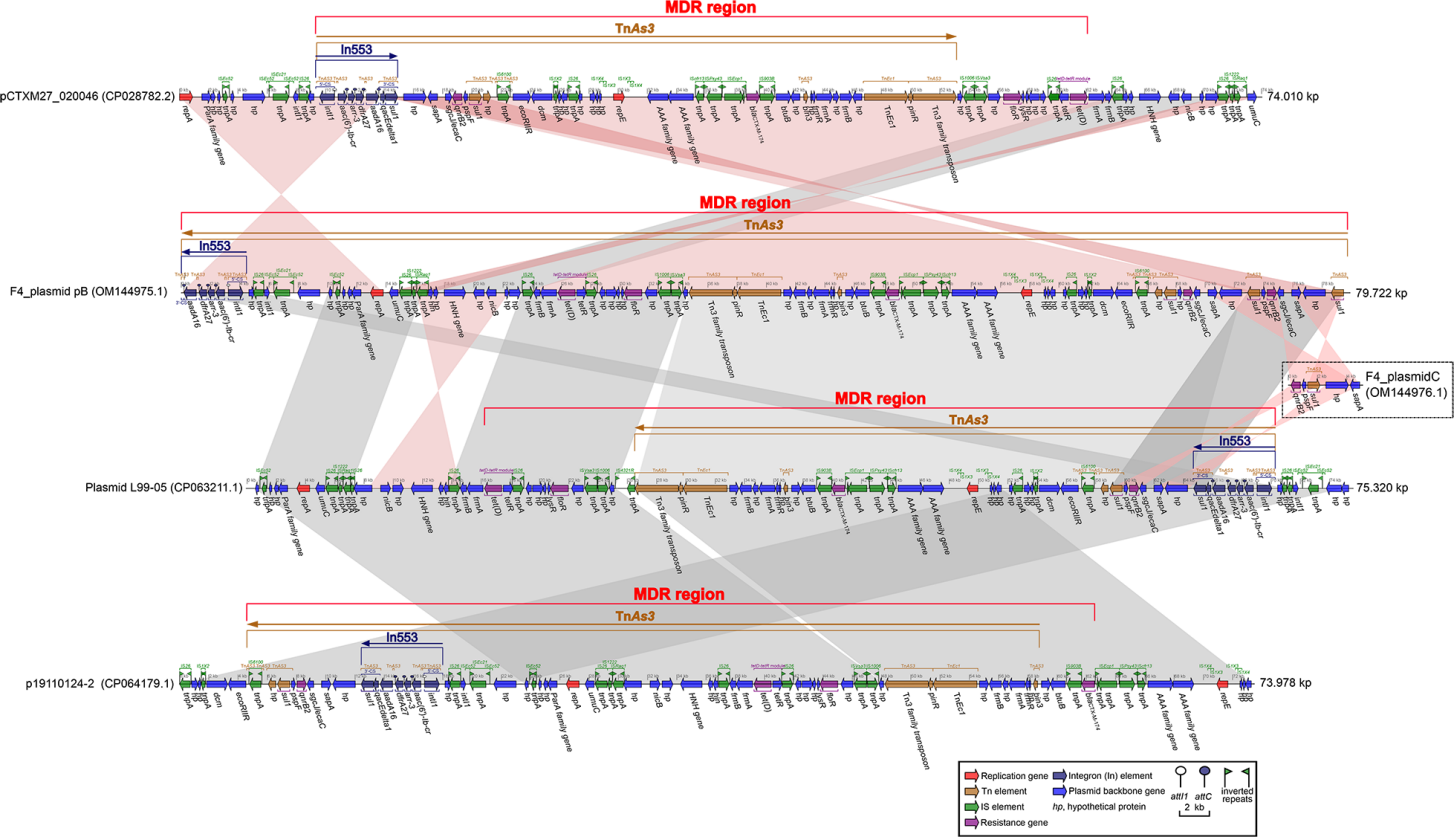
Comparison of the MDR regions from F4_plasmid pA and similar plasmids pMH15-269M_1, pHN111RT-1, pSZP4-9-2-tmexCD, and P1-tmexCD. The shadow represents >95% identity, while light blue represents the positive direction, and light pink refers to the opposite direction. The figure was created by the R package genoPlotR v0.8.11 software (http://genoplotr.r-forge.r-project.org/).

The profiles of these closely related plasmids are shown in **Table 2**. The identities and coverage rates between F4_plasmid pA and pMH15-269M_1, pHN111RT-1, pSZP4-9-2-tmexCD, and P1-tmexCD were all greater than 99% and 95%, respectively, with extremely high similarity. The lengths of the MDR regions from F4_plasmid pA, pMH15-269M_1, pHN111RT-1, pSZP4-9-2-tmexCD, and P1-tmexCD were estimated to be 65.8 kb, 64.3 kb, 66.6 kb, 63.1 kb, and 66.5 kb, respectively, where only an *aac(6’)-Ib* gene was outside the MDR region of each plasmid (**Figure S3**). The most significant common feature of these plasmids is that their MDR regions carry *tmexCD1-toprJ1*, *bla*_DHA-1_, and *bla*_CTX-M-127_, interpenetrated with Tn*As1*, Tn*5393*, and Tn*As3*, and intercalated with incomplete In641 (**Figure 2**). Tn*As1* was divided by Tn*5393* and Tn*As3*, which in turn was separated by In641 and a portion of Tn*5393* containing *tmexCD1-toprJ1*, the *mphE-msrE* module, and multiple insertion sequences (IS) including IS*26* (**Figure 2**). The common regions of Tn*As3* and Tn*5393* contained the RND-type efflux gene cluster *tmexCD1-toprJ1*, which was linked to the same IS*26* at both ends, forming an independent and basic structure as IS*26*-hp-IS*903B*-Tn*5393*-(*toprJ1-tmexD1-tmexC1*-*tnfxB1*)-hp-hp-IS*26* (**Figure 2**), which might have separate mobile properties, with *tnfxB1* acting as a plasmid-encoded transcriptional regulator for *tmexCD1-toprJ1*. In641 located on F4_plasmid pA and pMH15-269M_1 comprised the *estX*-*psp*-*aadA2*-*cmlA1*-*aadA1*- *qacH2* gene cassette with 5′-CS but lacking 3′-CS; In641 on pHN111RT-1 consisted of the *estX*-*psp*-*aadA2*-*cmlA1*-*aadA1* gene cassette with 5′-CS but no 3′-CS; In641 on pSZP4-9-2-tmexCD had only the *estX*-*psp*-*emrR* gene cassette, which had 5′-CS but lacked 3′-CS; and In641 on P1-tmexCD covered the e*stX*-*psp*-*aadA2*-*cmlA1*-*aadA1*- *emrR* gene cassette, with 5′-CS and a shortened 3′-CS (**Figure 2**). Tn*5393* was also compartmentalized by some elements, such as a gene array of IS*26*-hp-IS*903B*- Tn*5393*-(*toprJ1-tmexD1-tmexC1-tnfxB1*)-hp-hp-IS*26*, some IS, the *mphE-msrE* module, *psp* module, and some drug resistance genes, such as *bla*_DHA-1_, *aph(3’)-Ia*, *sul1*, and *qnB4* (**Figure 2**). Overall, F4_plasmid pA harbored mainly *tmexCD1-toprJ1* and *bla*_DHA-1_, accompanied by genetic intermixing of In641 with Tn*As1*, Tn*5393*, and Tn*As3*.

### Comparison of F4_plasmid pB with the closely related plasmids pCTXM27_020046, Plasmid L99-05, and p19110124-2, all harboring In553 and *bla*_CTX-M-174_

The profiles of F4_plasmid pB and closely related plasmids are shown in **Table 2**. The identities and coverage rates between F4_plasmid pB and pCTXM27_020046, Plasmid L99-05, and p19110124-2 were all greater than 99% and 92.5%, respectively, expressing extremely high similarity (**Table 2**).

The nearly 13.74 kb-long segment of pCTXM27_020046 from the *repA* gene to the partial 3′-CS of In553 (excluding *qacEdelta1* and *sul1*) was almost identical to the approximately 13.81 kb-long F4_plasmid pB from the 3′-CS remnant of incomplete In553 (without *qacEdelta1* and *sul1*) to the *repA* gene but in opposite directions (**Figure 1**). Similarly, an approximately 50.40 kb-long segment of pCTXM27_020046, from the *sul1* gene of In553 to IS*26* located at a site of approximately 64 kb, was almost identical to an approximately 47.80 kb-long segment of F4_plasmid pB, from IS26 located at a site of about 23 kb to the *sul1* gene located at site of nearly 73 kb, but in opposite directions (**Figure 1**).

When comparing F4_plasmid pB with Plasmid L99-05, the two plasmids were almost identical in structure and length, except for a small segment of sequence at the beginning of F4_plasmid pB that included In553, which was identical to the end of Plasmid L99-05 (**Figure 1**). When comparing Plasmid L99-05 with p19110124-2, the two plasmids were also nearly identical in structure and length, except that approximately a third of the final sequence length of p19110124-2 including In553 was the same as approximately a third of the length of the beginning of plasmid L99-05. F4_ plasmid pC contains five genes, two of which are the drug resistance genes *qnrB4* and *sul1*, and showed a high degree of sequence identity with the back-end sequence of F4_plasmid pB and a segment of Plasmid L99-05 located in front of In553 (**Figure 1**).

Structurally, Tn*As3* was divided into several parts by In553, some IS, and partial plasmid backbone genes located on F4_plasmid pB and its other closely related plasmids pCTXM27_020046, Plasmid L99-05, and p19110124-2, developing an intricate yet intersecting complex MDR region for each plasmid (**Figure 1**).

### Genetic context of *toprJ1-tmexCD1-tnfxB1* from F4_plasmid pA and closely related plasmids

By finely analyzing the MDR region in **Figure 2**, we discovered that IS*26* was present at both the front and back of *toprJ1-tmexCD1-tnfxB1*. IS*26*-mediated translocation is the most efficient and most likely to occur when a copy of IS*26* was involved (Harmer et al., 2014).

**Figure 3:**
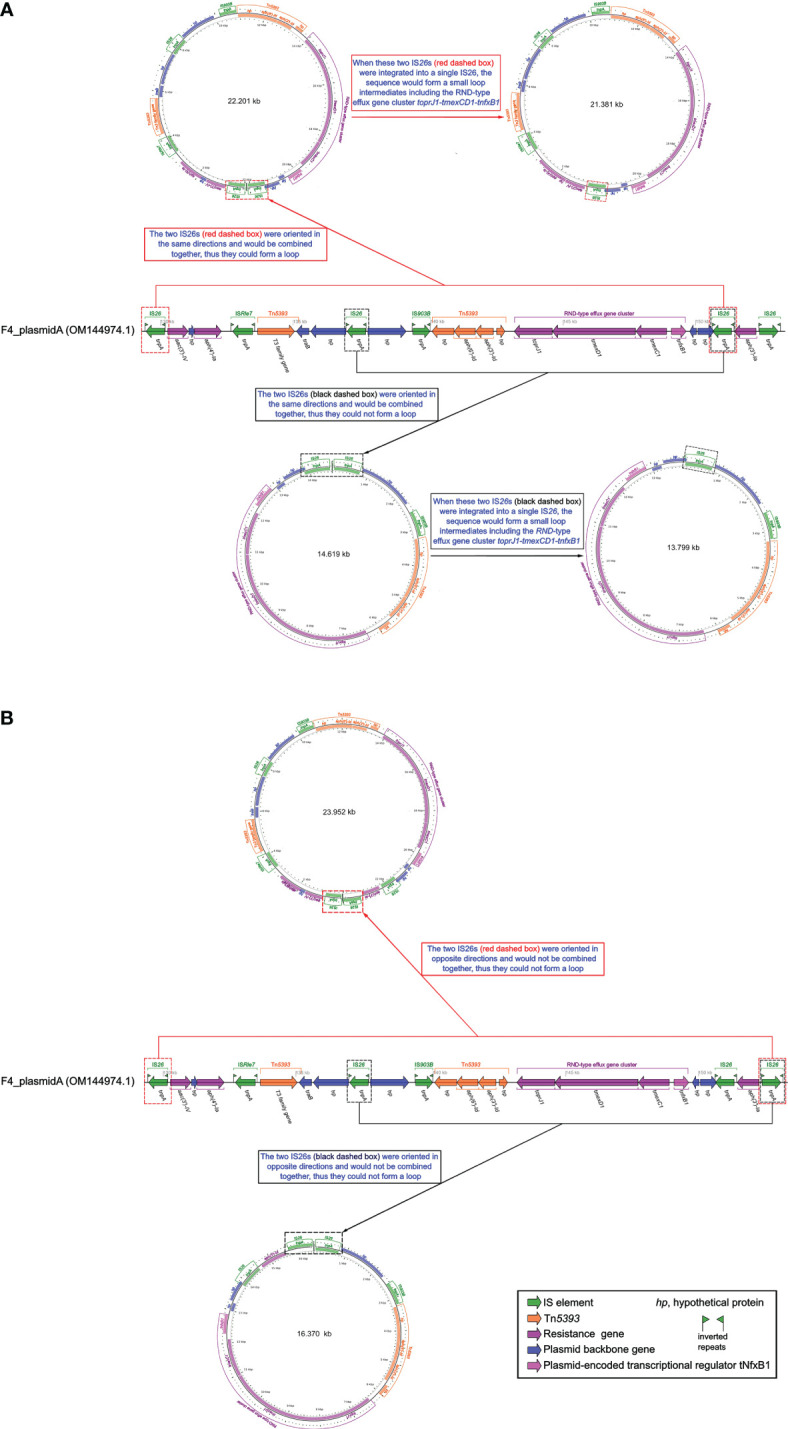
Schematic diagram of whether small loop intermediates could be formed by the integration of IS*26*s. **(A)** Schematic representation of a small loop intermediate that could be formed. **(B)** Schematic representation of a small loop intermediate that could not be formed. The diagram of F4_plasmid pA was created by the R package genoPlotR v0.8.11 software (http://genoplotr.r-forge.r-project.org/). The small loop diagrams were established using CGview v2.0.3 (https://github.com/paulstothard/cgview).

The IS*26*s of F4_plasmid pA and closely related plasmids pMH15-269M_1, pHN111RT-1, pSZP4-9-2-tmexCD, and P1-tmexCD were consistent, and these IS*26*s were localized to the gene arrays of IS*26*-hp-IS*903B*-Tn*5393*-(*toprJ1*-*tmexD1*- *tmexC1*-*tnfxB1*)-hp-IS*26* and IS*26*-*aac(3’)-IV*-hp-*aph(4’)-Ia*-IS*26*-hp-IS*903B*-Tn*5393* -(*toprJ1*-*tmexD1*-*tmexC1*-*tnfxB1*)-hp-hp-IS*26* (**Figure 2**). Although we had not yet been able to verify this experimentally, a similar case had been confirmed in the literature (Wan et al., 2021). Thus, we assumed that the above two structures could be integrated by IS*26* to form two circular intermediates with different sequence lengths (**Figure 3**), implying that these intermediates might also move independently, much like the characteristics of small plasmids. Taken together, this suggests that *tmexCD1*-*toprJ1* might have been transferred to different plasmids including the F4_plasmid pA and closely related plasmids via IS*26*-integrated loop intermediates, representing a novel kind of dispersal dissemination. For F4_plasmid pA, in addition to carrying *tmexCD1-toprJ1*, this plasmid also carried In641, Tn*As1*, Tn*As3*, and Tn*5393* intermingled with each other, which was more complex and posed a much greater clinical threat than pHN111RT-1 and pHN111WT-1 (Wan et al., 2021).

## Discussion

*tmexCD-toprJ*-positive bacteria are usually directly involved in multidrug resistance and can carry different drug resistance genes (Dong et al., 2022). The major functional *tmexCD* transporters have evolved into different isoforms. *tmexCD-toprJ* has a highly diverse genetic environment related to various mobile elements (Dong et al., 2022). If horizontal and vertical gene transfer of *tmexCD-toprJ* had occurred, it would likely have led to widespread clinical dissemination (Dong et al., 2022).

So far, *tmexCD-toprJ*-positive bacteria have originated largely in China from 2020, and have been occasionally discovered in Vietnam and other countries, provoking warnings of global spread (Hirabayashi et al., 2021). This may be related to the IS*26*-integrated loop intermediates described in this study (**Figure 3**). IS*26* could be transferred not only by a transposon consisting of two IS*26* and the related gene, but also by seamless transfer of the related gene to the end of the existing IS*26* (Harmer and Hall, 2015; Harmer and Hall, 2016). The efficiency of the second transfer was 60 times higher than that of the first transfer when the plasmid contained IS26 (Harmer et al., 2014). Furthermore, all transfer units, such as F4_plasmid pA and similar plasmids, carried the *traB* gene (**Figures 2**, **3**), which might accelerate transfer across species (Burbank and Van Horn, 2017).

The spread of the *tmexCD-toprJ* gene cluster involves ISs (such as IS*26* and IS*903B*), transposons (such as Tn*As1*, Tn*5393*, and Tn*As3*), integrons (such as In641), integration and conjugation elements (ICEs), and plasmids (**Figure 2**) (Sun et al., 2020; Wang Q. et al., 2021). These mobile genetic elements have contributed significantly to the development of bacterial antibiotic resistance. Incomplete Tn*As1*, Tn*5393*, and Tn*As3* with integron components were found in the *tmexCD1*-*toprJ1* gene clusters in the MDR regions of F4_plasmid pA and similar plasmids (**Figure 2**), and a complete or incomplete In641 appeared near the *tmexCD1*-*toprJ1* gene clusters; we speculate that these structures of the F4_plasmid pA and similar plasmids might have accompanied the spread of the *tmexCD1*-*toprJ1* gene cluster at an early stage and subsequently been disrupted and partitioned by other genetic mobile elements during the evolutionary process. Similarly, although F4_plasmid pB does not contain the *tmexCD1-toprJ1* gene cluster, incomplete Tn*As3* with In553 was also split from the MDR regions of F4_plasmid pB and similar plasmids, and complete or incomplete In553 appeared as one of the Tn*As3* components in these MDR regions (**Figure 3**). The presence of the *oqxAB* gene in the F4_chromosome increases the complexity of resistance to some antibiotics. A strain F4 carrying F4_plasmid pA with Tn*As1*, Tn*5393*, Tn*As3*, In641, *tmexCD1*-*toprJ1*, and *bla*_DHA-1_ (**Figure 2**), alongside F4_plasmid pB with Tn*As3*, In641, and *bla*_CTX-M-174_ (**Figure 1**) and the F4_chromosome bearing the *oqxAB* gene (**Figure S1**), would be extremely challenging for clinical prevention and treatment. Mobilization and dissemination of bacterial plasmids carrying *toprJ1-tmexCD1* and incorporating IS*26* as a mediator would be more dangerous in hospitals.

Overexpression of *oqxAB*, the RND-type efflux pump gene on the chromosome, plays an essential role in tigecycline resistance (Sheng et al., 2014; Bialek-Davenet et al., 2015). Strain F4 was highly resistant to 22 routinely used antibiotics and antibiotic + enzyme inhibitors, including tigecycline, and this resistance was directly associated with the *oqxAB* gene localized on the F4_chromosome (**Figure S1**) and *tmexCD1*-*toprJ1* on plasmid A (**Table 1**). The high resistance to β-lactams and β-lactams + enzyme inhibitors, especially the novel combination of cefoperazone + avibactam, was directly related to *bla*_DHA-1_ on F4_plasmid pA and *bla*_CTX-M-174_ on F4_plasmid pB (**Table 1**), so was cephalexin resistance (**Table 1**). Overall, strain F4 expressed to resistance to Aminoglycoside, β-lactams antibiotics, β-lactams antibiotics + enzyme inhibitors, Cephalexin, Fluoroqinolones, Tetracycline, Glycylcycline (tigecycline), Phenicol, Sulfonamide, Macrolide and Rifamycin (**Table 1**). The emergence of the plasmid-mediated multidrug resistance gene cluster *tmexCD1*-*toprJ1* decreases susceptibility to many clinically important antimicrobial drugs and constitutes a serious problem of multidrug resistance, which is the most troublesome issue in the human clinical setting. It’s worth noting that if *tnfxB* function was absent, overexpression of *tmexCD1*-*toprJ1* could enhanced resistance to tetracyclines, fluoroquinolones, cephalosporins, macrolides, and chloramphenicol (**Table 1**), and in the presence of upstream *tnfxB*, *tmexCD1*-*toprJ1* would not been affected the resistance level (Lv et al., 2020). Structural differences in the composition of resistance genes between F4_plasmid pA, F4_plasmid pB and F4_plasmid pC were responsible for the differences in antibiotic resistance among strains F4 (**Table 1**).

## Conclusion

It has risen to such a terrible extent that there are almost no effective antibiotics available, and its spread and prevalence should be effectively prevented for strain F4 and related strains, because of the intermingling of In641 with Tn*As1*, Tn*5393*, and Tn*As3* in F4_ plasmid pA and closely related plasmids carrying *tmexCD1-toprJ1* and *bla*_DHA-1_, and the structural overlaps of In553 and Tn*As3* in F4_plasmid pB and closely related plasmids bearing *bla*_CTX-M-147_, together with the *oqxAB* genes located on the F4 chromosome.

## Data availability statement

The datasets presented in this study can be found in online repositories. The names of the repository/repositories and accession number(s) can be found below: https://www.ncbi.nlm.nih.gov/genbank/, The GenBank accession numbers of the F4_chromosome, F4_plasmid pA, F4_plasmid pB, and F4_plasmid pC are CP090397.1, OM144974.1, OM144975.1, and OM144976.1, respectively.

## Ethics statement

This study was approved by the Ethics Committee of Taizhou Municipal Hospital, Zhejiang, China, and written informed consent was obtained from each of the participants in accordance with the Declaration of Helsinki. The rights of the research subjects were protected throughout, and we confirm that this study was conducted in our hospital. The use of human specimens and all related experimental protocols were approved by the Committee on Human Research of the indicated institutions, and the protocols were carried out in accordance with approved guidelines.

## Author contributions

YQ: Data curation, Formal Analysis, Funding acquisition, Investigation, Methodology, Project administration, Resources, Supervision, Validation, Writing – review & editing, Software. WW: Data curation, Formal Analysis, Investigation, Methodology, Project administration, Resources, Software, Supervision, Validation, Writing – review & editing. QL: Data curation, Formal Analysis, Investigation, Methodology, Project administration, Resources, Software, Supervision, Validation, Writing – review & editing. JQ: Data curation, Formal Analysis, Investigation, Methodology, Project administration, Resources, Supervision, Validation, Writing – review & editing. DW: Data curation, Formal Analysis, Investigation, Methodology, Project administration, Resources, Supervision, Validation, Writing – review & editing, Conceptualization, Funding acquisition, Writing – original draft. LM: Writing – review & editing, Data curation, Formal Analysis, Investigation, Methodology, Project administration, Resources, Software, Supervision, Validation.

## Supplementary material

Supplementary Figure 1 Circos plot of the F4_chromosome and localization of antibiotic resistance genes. **(A)** F4_chromosome of 5.249104 Mbp in length showing the location of the *oqxAB*, *bla*_CTX-M-27_, and *bla*_SHV-106_ genes. **(B)** Linear plots of structural features for the antibiotic resistance genes *oqxAB*, *bla*_CTX-M-27_, and *bla*_SHV-106_. The Circos plot of F4_chromosome was established using CGview v2.0.3 (https://github.com/paulstothard/cgview). The locations of the antibiotic resistance genes were drawn manually using Inkscape 0.48.1 (https://inkscape.org/en). **Figure S1B** was created by the R package genoPlotR v0.8.11 software (http://genoplotr.r-forge.r-project.org/) with hand finishing.

Supplementary Figure 2 Circos plots of F4_plasmid pA **(A)**, F4_plasmid pB **(B)**, and F4_plasmid pC **(C)**. The figure was established using CGview v2.0.3 (https://github.com/paulstothard/cgview).

Supplementary Figure 3 Comparison of F4_plasmid pA with related plasmids pMH15-269M_1, pHN111RT-1, pSZP4-9-2-tmexCD, and P1-tmexCD. The shadow represents >95% identity, while light blue represents the positive direction, and light pink refers to the opposite direction. The figure was created by the R package genoPlotR v0.8.11 software (http://genoplotr.r-forge.r-project.org/).
