# Supplementary figures and images for: Occurrence and characterization of plasmids carrying tmexCD1-toprJ1, bla DHA-1, and bla CTX-M-127, in clinical Klebsiella pneumoniae strains
Source: Front Cell Infect Microbiol. 2023 Oct 13;13:1260066. doi: 10.3389/fcimb.2023.1260066 (PMC10611489; doi:10.3389/fcimb.2023.1260066)

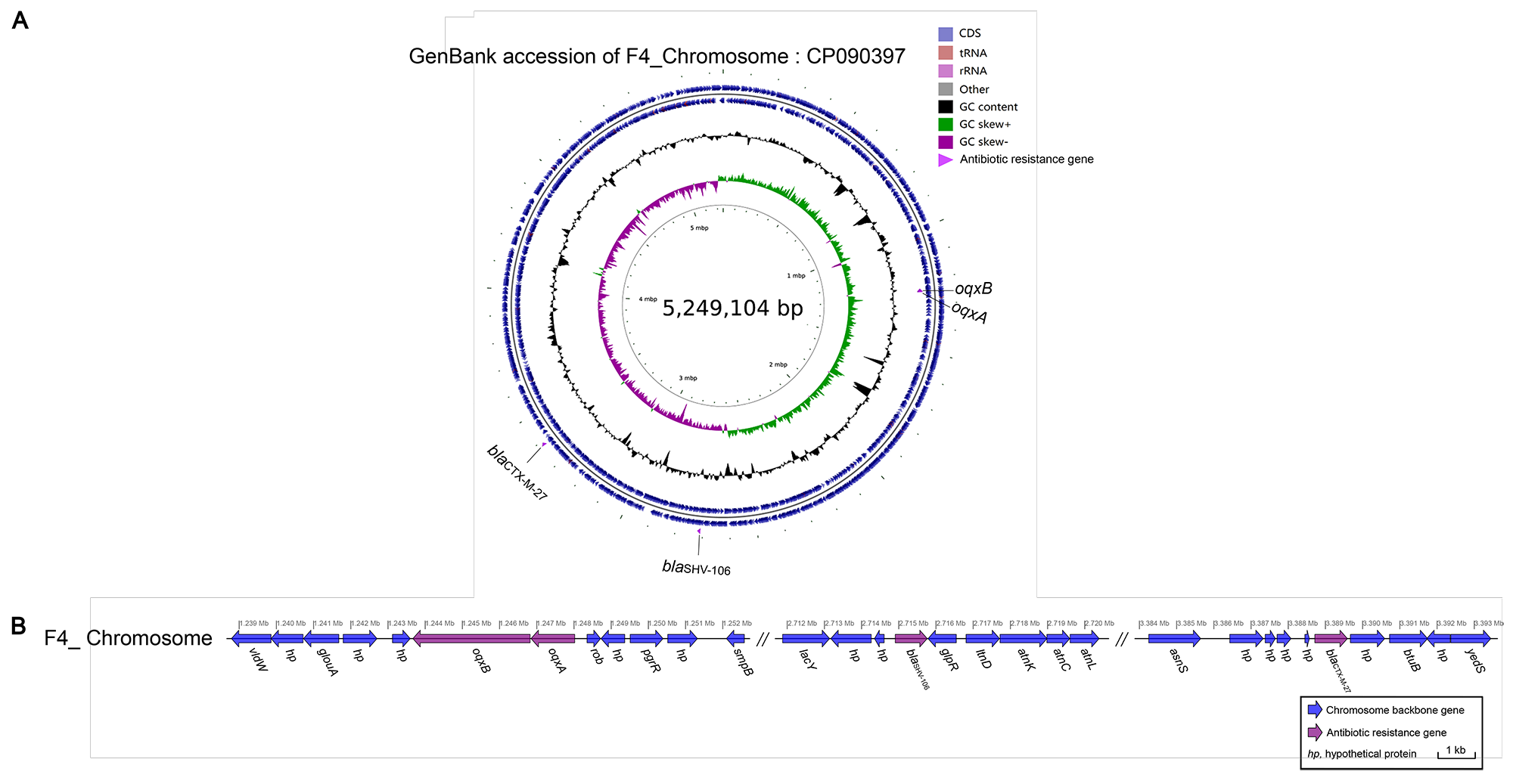

Supplement: Supplementary Figure 1 — Circos plot of the F4_chromosome and localization of antibiotic resistance genes. (A) F4_chromosome of 5.249104 Mbp in length showing the location of the oqxAB, bla CTX-M-27, and bla SHV-106 genes. (B) Linear plots of structural features for the antibiotic resistance genes oqxAB, bla CTX-M-27, and bla SHV-106. The Circos plot of F4_chromosome was established using CGview v2.0.3 (https://github.com/paulstothard/cgview). The locations of the antibiotic resistance genes were drawn manually using Inkscape 0.48.1 (https://inkscape.org/en). Figure S1B was created by the R package genoPlotR v0.8.11 software (http://genoplotr.r-forge.r-project.org/) with hand finishing. [file Image_1.tif]

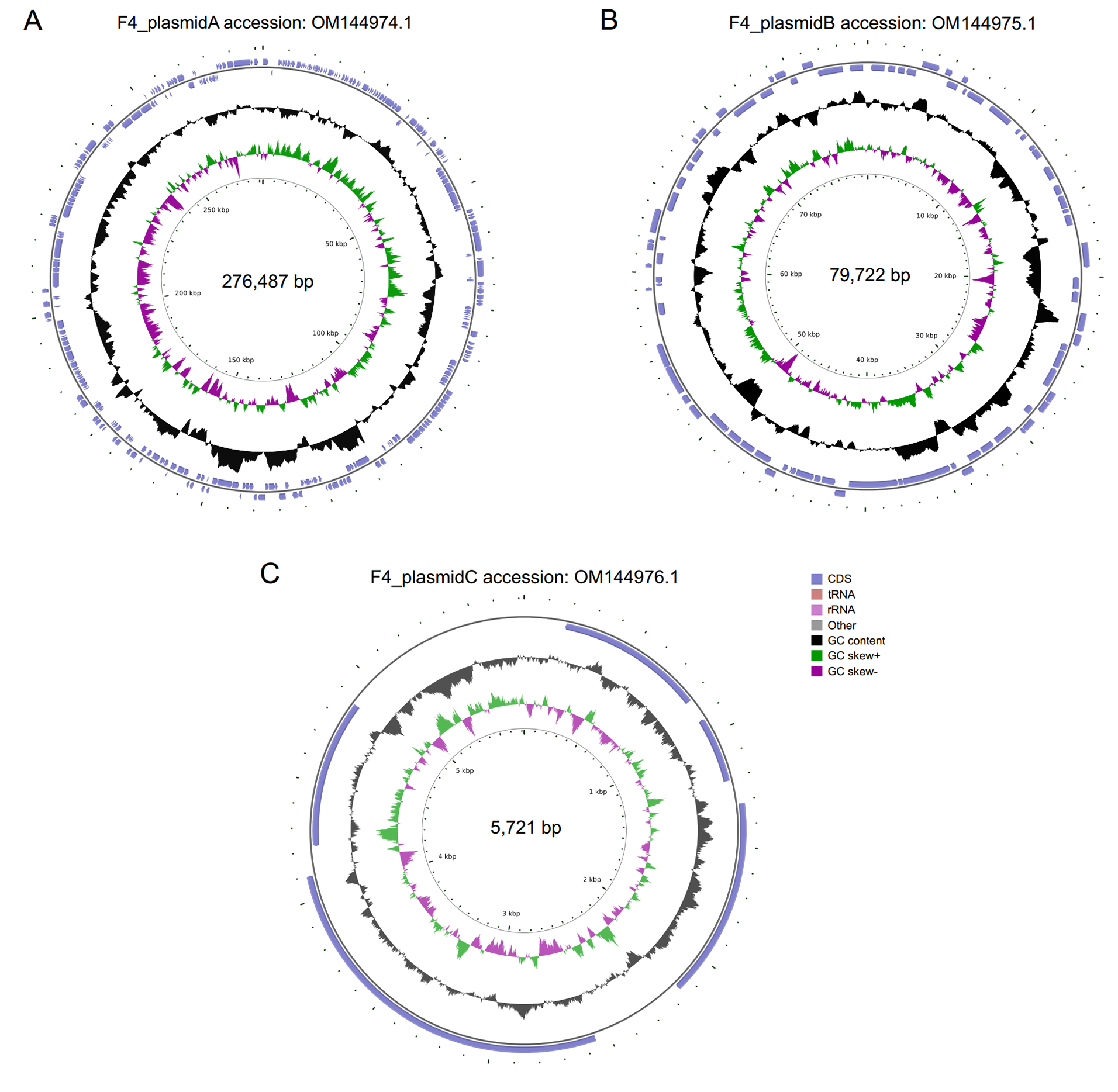

Supplement: Supplementary Figure 2 — Circos plots of F4_plasmid pA (A), F4_plasmid pB (B), and F4_plasmid pC (C). The figure was established using CGview v2.0.3 (https://github.com/paulstothard/cgview). [file Image_2.tif]

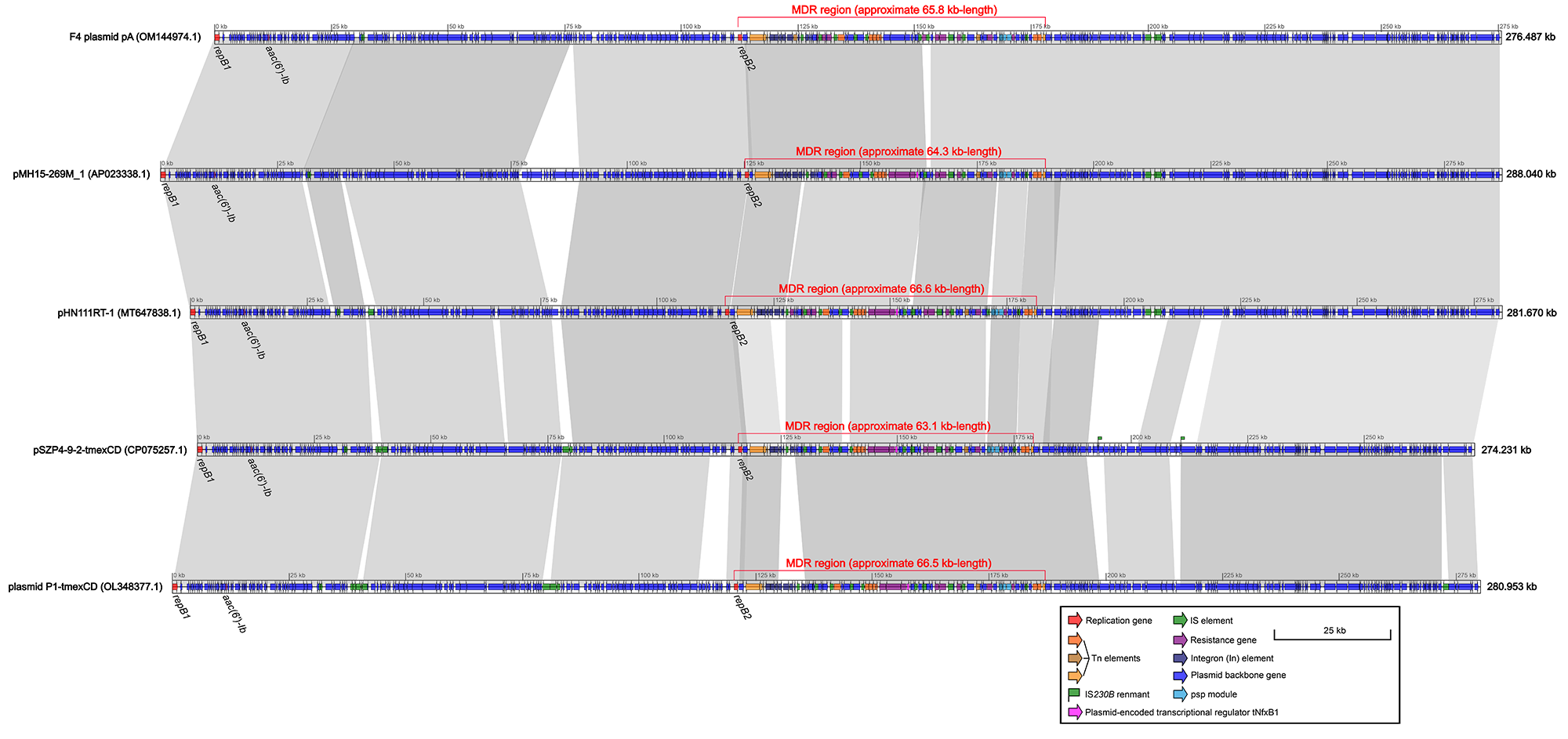

Supplement: Supplementary Figure 3 — Comparison of F4_plasmid pA with related plasmids pMH15-269M_1, pHN111RT-1, pSZP4-9-2-tmexCD, and P1-tmexCD. The shadow represents >95% identity, while light blue represents the positive direction, and light pink refers to the opposite direction. The figure was created by the R package genoPlotR v0.8.11 software (http://genoplotr.r-forge.r-project.org/). [file Image_3.tif]
