# Supplementary material for: Occurrence and characterization of plasmids carrying tmexCD1-toprJ1, bla DHA-1, and bla CTX-M-127, in clinical Klebsiella pneumoniae strains
Source: Front Cell Infect Microbiol. 2023 Oct 13;13:1260066. doi: 10.3389/fcimb.2023.1260066 (PMC10611489; doi:10.3389/fcimb.2023.1260066)
Supplement: Supplementary file 4 [file Table_1.docx]

| **Table S1 Main genes of MDR region for F4_plasmid pA and related plasmids** | | | | |
| --- | --- | --- | --- | --- |
| **Gene type or**  **Gene function** | **F4_plasmid pA** | **pMH15-269M_1** | **pHN111RT-1** | **pSZP4-9-2-tmexCD** |
| Duplication gene | *repB2* | *repB2* | *repB2* | *repB2* |
| Transposon element | Tn*As1* | Tn*As1* | Tn*As1* | Tn*As1* |
| In641 | *intI1* | *intI1* | *intI1* | *intI1* |
|  | *estX* | *estX* | *estX* | *estX* |
|  | *psp* | *psp* | *psp* | *psp* |
|  | *aadA2* | *aadA2* | *aadA2* | – |
|  | *cmlA1* | *cmlA1* | – | – |
|  | – | – | – | – |
|  | – | – | – | *emrE* |
|  | *qacH2* | *qacH2* | – | – |
| Insertion element | IS*406* | IS*406* | IS*406* | IS*406* |
| Sulfonamide | *sul3* | *sul3* | *sul3* | *sul3* |
| Macrolide | *mefB* | *mefB* | *mefB* | *mefB* |
| Insertion element | IS*26* | IS*26* | IS*26* | IS*26* |
| Insertion element | IS*Rle7* | IS*Rle7* | IS*Rle7* | IS*Rle7* |
| Transposon element | Tn*5393* | Tn*5393* | Tn*5393* | Tn*5393* |
| Insertion element | IS*26* | IS*26* | IS*26* | IS*26* |
| Insertion element | IS*903B* | IS*903B* | IS*903B* | IS*903B* |
| Aminoglycoside | *aph(6')-Id* | *aph(6')-Id* | *aph(6')-Id* | *aph(6')-Id* |
| Aminoglycoside | *aph(3')-Id* | *aph(3')-Id* | *aph(3')-Id* | *aph(3')-Id* |
| RND-type  effux genes cluster | *toprJ1* | *toprJ1* | *toprJ1* | *toprJ1* |
|  | *tmexD1* | *tmexD1* | *tmexD1* | *tmexD1* |
|  | *tmexC1* | *tmexC1* | *tmexC1* | *tmexC1* |
|  | *tnfxB1* | *tnfxB1* | *tnfxB1* | *tnfxB1* |
| Insertion element | IS*26* | IS*26* | IS*26* | IS*26* |
| Aminoglycoside | *aph(3')-Ia* | – | – | – |
| Insertion element | IS*26* | IS*26* | IS*26* | IS*26* |
| Macrolide | *mphE* | *mphE* | *mphE* | *mphE* |
| Macrolide | *msrE* | *msrE* | *msrE* | *msrE* |
| Insertion element | IS*Ec29* | IS*Ec29* | IS*Ec29* | IS*Ec29* |
| Aminoglycoside | *rmtB* | *rmtB* | *rmtB* | *rmtB* |
| Insertion element | IS*Ec35* | IS*Ec35* | IS*Ec35* | IS*Ec35* |
| Insertion element |  | – | IS*Vsa35* | – |
| Sulfonamide | *sul1* | *sul1* | *sul1* | *sul1* |
| β-lactams | *bla*DHA-1 | *bla*DHA-1 | *bla*DHA-1 | *bla*DHA-1 |
| Insertion element | – | – | IS*1A* | – |
| Fluoroqinolones | *qnrB4* | *qnrB4* | *qnrB4* | *qnrB4* |
| Insertion element | IS*1R* | IS*1R* | IS*1R* | IS*1R* |
| Aminoglycoside | *aph(3')-Ib* | *aph(3')-Ib* | *aph(3')-Ib* | *aph(3')-Ib* |
| Aminoglycoside | *aph(6')-Id* | *aph(6')-Id* | *aph(6')-Id* | *aph(6')-Id* |
