# Supplementary material for: Occurrence and characterization of plasmids carrying tmexCD1-toprJ1, bla DHA-1, and bla CTX-M-127, in clinical Klebsiella pneumoniae strains
Source: Front Cell Infect Microbiol. 2023 Oct 13;13:1260066. doi: 10.3389/fcimb.2023.1260066 (PMC10611489; doi:10.3389/fcimb.2023.1260066)
Supplement: Supplementary file 5 [file Table_2.docx]

| **Table S2 Main genes for F4_plasmid pB and related plasmids** | | | | |
| --- | --- | --- | --- | --- |
| **Gene type or**  **Gene function** | **F4_plasmid pB** | **pCTXM27_020046** | **Plasmid L99-05** | **p19110124-2** |
| In553 | – | *sul1* | *sul1* | *sul1* |
|  | – | *qacEdelta1* | *qacEdelta1* | *qacEdelta1* |
|  | *aadA16* | *aadA16* | *aadA16* | *aadA16* |
|  | *dfrA27* | *dfrA27* | *dfrA27* | *dfrA27* |
|  | *arr-3* | *arr-3* | *arr-3* | *arr-3* |
|  | *aac(6')-Ib-cr* | *aac(6')-Ib-cr* | *aac(6')-Ib-cr* | *aac(6')-Ib-cr* |
|  | *intI1* | *intI1* | *intI1* | *intI1* |
| Insertion element | IS*26* | IS*26* | IS*26* | IS*26* |
| In0 | *intI1* | *intI1* | – | *intI1* |
| Insertion element | IS*Ec21* | IS*Ec21* | IS*Ec21* | IS*Ec21* |
| Insertion element | IS*Ec52* | IS*Ec52* | IS*Ec52* | IS*Ec52* |
| Duplication gene | *repA* | *repA* | *repA* | *repA* |
| Insertion element | IS*26* | IS*26* | IS*26* | IS*26* |
| Insertion element | IS*1222* | IS*1222* | IS*1222* | IS*1222* |
| Insertion element | IS*Raq1* | IS*Raq1* | IS*Raq1* | IS*Raq1* |
| Insertion element | IS*26* | IS*26* | IS*26* | IS*26* |
| *tetD*-*tetR* modual | *tetD* | *tetD* | *tetD* | *tetD* |
|  | *tetR* | *tetR* | *tetR* | *tetR* |
| Insertion element | IS*26* | IS*26* | IS*26* | IS*26* |
| Chloramphenicol | *floR* | *floR* | *floR* | *floR* |
| Insertion element | IS*1006* | IS*1006* | IS*1006* | IS*1006* |
| Insertion element | IS*Vsa3* | IS*Vsa3* | IS*Vsa3* | IS*Vsa3* |
| Transposon element | Tn*As3* | Tn*As3* | Tn*As3* | Tn*As3* |
| Transposon element | Tn*Ec1* | Tn*Ec1* | Tn*Ec1* | Tn*Ec1* |
| Insertion element | IS*903B* | IS*903B* | IS*903B* | IS*903B* |
| β-lactams | *bla*CTX-M-174 | *bla*CTX-M-174 | *bla*CTX-M-174 | *bla*CTX-M-174 |
| Insertion element | IS*Ecp1* | IS*Ecp1* | IS*Ecp1* | IS*Ecp1* |
| Insertion element | IS*Psy43* | IS*Psy43* | IS*Psy43* | IS*Psy43* |
| Insertion element | IS*Cfr13* | IS*Cfr13* | IS*Cfr13* | IS*Cfr13* |
| Duplication gene | *repE* | *repE* | *repE* | *repE* |
| Insertion element | IS*26* | IS*26* | IS*26* | IS*26* |
| Insertion element | IS*1X2* | IS*1X2* | IS*1X2* | IS*1X2* |
| Insertion element | IS*6100* | IS*6100* | IS*6100* | IS*6100* |
| Sulfonamide | *sul1* | *sul1* | *sul1* | *sul1* |
| Fluoroqinolones | *qnrB2* | *qnrB2* | *qnrB2* | *qnrB2* |
| Sulfonamide | *sul1* | *sul1* | *sul1* | *sul1* |
| Fluoroqinolones | *qnrB2* | *qnrB2* | *qnrB2* | *qnrB2* |
| Sulfonamide | *sul1* | *sul1* | *sul1* | *sul1* |
