# Supplementary material for: Occurrence and characterization of plasmids carrying tmexCD1-toprJ1, bla DHA-1, and bla CTX-M-127, in clinical Klebsiella pneumoniae strains
Source: Front Cell Infect Microbiol. 2023 Oct 13;13:1260066. doi: 10.3389/fcimb.2023.1260066 (PMC10611489; doi:10.3389/fcimb.2023.1260066)
Supplement: Supplementary file 6 [file Table_3.docx]

| **Table S3 Main genes for F4_plasmid pC and related plasmids** | | | |
| --- | --- | --- | --- |
| **Gene type or**  **Gene function** | **F4_plasmid pC** | **F4_plasmid pB** | **Plasmid L99-05** |
| Fluoroqinolones | *qnrB2* | *qnrB2* | *qnrB2* |
| Sulfonamide | *sul1* | *sul1* | *sul1* |
| Sulfonamide | – | *sul1* | *sul1* |
